# The Molecular Mechanism of Human Voltage-Dependent Anion Channel 1 Blockade by the Metallofullerenol Gd@C_82_(OH)_22_: An In Silico Study

**DOI:** 10.3390/biom12010123

**Published:** 2022-01-12

**Authors:** Xiuxiu Wang, Nan Yang, Juan Su, Chenchen Wu, Shengtang Liu, Lei Chang, Leigh D. Plant, Xuanyu Meng

**Affiliations:** 1 State Key Laboratory of Radiation Medicine and Protection, Institute of Quantitative Biology and Medicine, Collaborative Innovation Center of Radiation Medicine of Jiangsu Higher Education Institutions, School of Radiation Medicine and Protection, Soochow University, Suzhou 215123, China; 20194020004@stu.suda.edu.cn (X.W.); 20194220051@stu.suda.edu.cn (N.Y.); 20194220060@stu.suda.edu.cn (J.S.); ccwu@stu.suda.edu.cn (C.W.); changlei@suda.edu.cn (L.C.); 2 Department of Pharmaceutical Sciences, Bouvé College of Health Sciences, Northeastern University, 360 Huntington Avenue, Boston, MA 02115, USA

**Keywords:** molecular dynamics simulations, Gd-fullerenol, VDAC1, PMF, nanodrug

## Abstract

The endohedral metallofullerenol Gd@C_82_(OH)_22_ has been identified as a possible antineoplastic agent that can inhibit both the growth and metastasis of cancer cells. Despite these potentially important effects, our understanding of the interactions between Gd@C_82_(OH)_22_ and biomacromolecules remains incomplete. Here, we study the interaction between Gd@C_82_(OH)_22_ and the human voltage-dependent anion channel 1 (hVDAC1), the most abundant porin embedded in the mitochondrial outer membrane (MOM), and a potential druggable target for novel anticancer therapeutics. Using in silico approaches, we observe that Gd@C_82_(OH)_22_ molecules can permeate and form stable interactions with the pore of hVDAC1. Further, this penetration can occur from either side of the MOM to elicit blockage of the pore. The binding between Gd@C_82_(OH)_22_ and hVDAC1 is largely driven by long-range electrostatic interactions. Analysis of the binding free energies indicates that it is thermodynamically more favorable for Gd@C_82_(OH)_22_ to bind to the hVDAC1 pore when it enters the channel from inside the membrane rather than from the cytoplasmic side of the protein. Multiple factors contribute to the preferential penetration, including the surface electrostatic landscape of hVDAC1 and the unique physicochemical properties of Gd@C_82_(OH)_22_. Our findings provide insights into the potential molecular interactions of macromolecular biological systems with the Gd@C_82_(OH)_22_ nanodrug.

## 1. Introduction

Gadolinium endohedral fullerenol Gd@C_82_(OH)_22_ is a nanomaterial that was initially designed as a contrast agent for magnetic resonance imaging (MRI) [1,2]. By encapsulating naked Gd^3+^ in a stable fullerene cage, Gd@C_82_(OH)_22_ preserves the high proton relaxivity of Gd^3+^ while reducing its toxicity. The biocompatibility of Gd@C_82_(OH)_22_ was augmented by subsequent modifications at hydroxyl groups on the surface of the carbon cage [3,4]

In addition to its benefits in contrast imaging, Gd@C_82_(OH)_22_ is a potential antineoplastic agent that can inhibit tumor growth in liver, kidney, pancreas, lung, and breast tissue *in vivo* with high efficiency and low toxicity [3,5,6,7,8,9,10,11,12]. Gd@C_82_(OH)_22_ inhibits tumor growth and metastasis via numerous mechanisms, including reducing oxidative stress and reactive oxygen species (ROS) formation in both healthy and tumor tissues [9,13,14]; suppressing the expression of matrix metalloproteinase (MMP) enzymes; reducing the catalytic activity of MMP enzymes [12,15,16]; regulating the immune response [7,11,17,18,19]; and reducing tumor nutrient supply by inhibiting tumor angiogenesis and tumor-vessel density [3].

Despite the apparent multidimensional benefits of Gd@C_82_(OH)_22_, little is known about the molecular mechanisms that mediate its antineoplastic effects. Adopting an in silico approach, our group identified the binding interface for Gd@C_82_(OH)_22_ on MMP-9, revealing the molecular mechanism by which Gd@C_82_(OH)_22_ inhibits enzyme activity. This insight provided a framework for studying how MMP-9 inhibition can lead to the imprisonment of cancer cells by (i) reducing tumor angiogenesis, (ii) preserving the extracellular matrix, and (iii) reducing subsequent metastasis [12]. Additional in silico studies combined with experimental data further expanded on the imprisonment mechanism, showing that Gd@C_82_(OH)_22_ mediates a “bridge-like” mode of interaction and signaling between tumor necrosis factor (TNFα) and tumor necrosis factor receptor 2 (TNFR2) [18].

When compared with conventional pharmacophores (usually small organic molecules), nanoparticles such as Gd@C_82_(OH)_22_ can be finely manipulated via chemical modification with a diverse array of functional groups to increase the efficacy of interactions with target bio-macromolecules (e.g., hydrogen bonding; electrostatic, hydrophobic, and hydrophilic interactions) [3,20,21,22,23,24]. This physicochemical and structural diversity implies high-level complexity and the potential for a multiplicity of Gd@C82(OH)22 interactions at the subcellular level, which deserves further investigation. In fact, several previous studies have shown that Gd@C82(OH)22 may potentially influence cellular signaling via direct interactions with WW and SH3 protein domains [25,26]. Both domains can mediate highly promiscuous protein–protein interactions and act as mediators commonly observed in signaling and regulatory pathways. Thus, the presence of Gd@C_82_(OH)_22_ may interfere with native protein–protein interactions to modulate both pathophysiological and physiological cell signaling pathways. Our previous studies also indicated that Gd@C_82_(OH)_22_ nanoparticles may selectively bind to specific domains on the cytochrome enzyme CYP2C8 [27], which has implications for enzyme activity.

**Figure 1:**
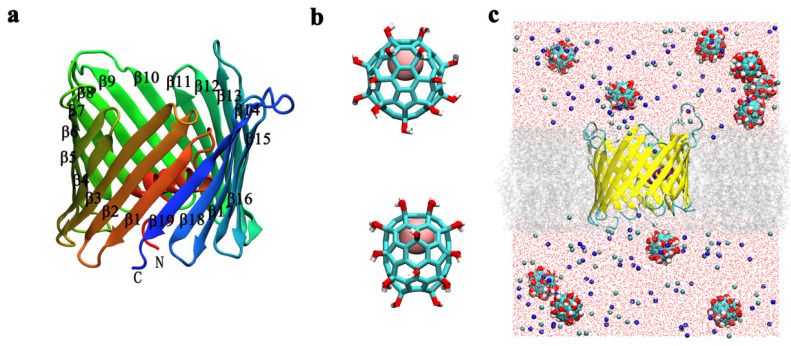
(**a**) The NMR solution structure of the hVDAC1 protein. The protein is illustrated with ribbon colors representing the transition from the N-terminus (red) to the C-terminus (blue). Human VDAC1 comprises a nine-residue-long N-terminal loop, an α-helix, and nineteen β-strands that form a circular bucket (β-barrel) with the N-terminal loop and helix embedded in the hollow lumen. (**b**) The structure of the Gd@C_82_(OH)_22_ molecule. The encaged Gd^3+^ ion is shown as a pink sphere, and the fullerenol cage is represented as sticks. (**c**) The initial in silico system setup. The Gd@C_82_(OH)_22_ molecule is shown with vdW sphere; hVDAC1 is shown with a yellow ribbon and colored according to the secondary structure elements. The protein is embedded in the lipid bilayer represented by grey lines. Blue and cyan balls represent potassium and chloride ions, respectively. The red dots are the oxygen atoms of water molecules.

In the present study, we focus on the interaction between Gd@C_82_(OH)_22_ and the voltage-dependent anion channel (VDAC), the most abundant channel protein embedded in the mitochondrial outer membrane (MOM) [28,29]. The VDAC protein family includes three isomers—VDAC1, VDAC2, and VDAC3—of which VDAC1 is the most abundant. Functional and structural studies show that VDAC1 forms a porelike structure with a β-barrel architecture composed of 19 β-strands with an N-terminal α-helix located horizontally midway within the pore (Figure 1a) [28,30,31]. VDAC1 is a gatekeeper for mitochondrial transmembrane flux and, thereby, plays a role in mitochondrial homeostasis by controlling metabolite and ion distribution between the cytosol and the interior of the organelle [30,32,33,34,35]. For this reason, VDAC1 is essential for the regulation of apoptosis [36,37,38,39]. High densities of VDAC1 protein in the MOM are proposed to form interactions with hundreds of protein partners, regulating mitochondrial function and the subsequent role of these organelles in diseases, including cancer [40,41,42].

Since VDAC1 is a potential druggable target for antineoplastic therapies, we sought to determine if the channel could interact with the anticancer nanodrug Gd@C_82_(OH)_22_. Here, we utilize all-atom molecular dynamics (MD) simulations together with free energy calculations to investigate the interactions between Gd@C_82_(OH)_22_ and the human isoform of the channel hVDAC1. Our results indicate that Gd@C_82_(OH)_22_ can penetrate the lumen of hVDAC1 from both sides of the MOM and block the pore. Blockage from the inside of the MOM (abbreviated as inside of membrane, IM) is energetically preferable to that from the outside of the MOM (abbreviated as outside of membrane, OM); further, both blocked-binding modes are energetically more favorable than the unblocked binding modes observed in the MD simulations. These results imply that Gd@C_82_(OH)_22_ may perturb the activity of the mitochondrial porin protein hVDAC1 and interfere with the biological functions of the mitochondria.

## 2. Methods

### 2.1. Molecular Dynamics Simulation

An atomic structure model of the hVDAC1 protein was obtained from the Protein Data Bank (PDB ID: 2jk4). The protein was embedded in a pre-equilibrated bilayer of the dimyristoylphosphatidylcholine (DMPC) [43] phospholipid bilayer, which was generated by CHARMM-GUI v1.7 [44] (http://www.charmm-gui.org, accessed on 24 November 2021). The hVDAC1 and bilayer were solvated in a rectangle box size of 10.032 × 10.032 × 12.000 nm^3^. Ten Gd@C_82_(OH)_22_ molecules were initially distributed in the simulation box at least 10 Å away from the protein. Please note that the simulation concentration of Gd@C_82_(OH)_22_ molecules used is much higher than that in the experiments to facilitate the sampling of the molecules within the limited simulation time and to mimic their aggregation behavior [45]. The simulation box (Figure 1c) was filled with TIP3P water molecules [46] and 150-mM KCl solution to mimic physiological conditions. The CHARMM36 force field [47] was used for the protein, lipids, and ions. The force field parameters of Gd@C_82_(OH)_22_ were obtained based on density functional theory (DFT) calculations, as described in our previous study [12]. VMD programs [48] were used for visualizing the simulation results.

MD simulations were performed using the GROMACS software package [49]. Simulated systems were kept at a temperature of 300 K using the v-rescale thermostat [50]. A pressure of 1 atm was coupled to the semi-isotropic (X + Y, Z) directions of the system using the Parrinello–Rahman algorithm [51]. Periodic boundary conditions (PBC) were applied in all three directions. The long-range electrostatic interactions were handled with the particle mesh Ewald (PME) method [52] with a cutoff distance of 1.0 nm. The van der Waals (vdW) interactions were computed at 1.0 nm cutoff. Water geometry was constrained by the SETTLE algorithm [53] and solute hydrogen bonds were constrained to their equilibrium values by employing the LINCS algorithm [54]. Each system was subjected to 2000 steps of energy minimization using the steepest descent method, followed by a 0.1-ns equilibration in which position restraints were added to protein-heavy atoms and the Gd@C_82_(OH)_22_ molecules with a constant force of 1000 kJ mol^−1^ nm^−2^. Nine independent simulations were conducted, each lasting 100–200 ns. The time step was 2.0 fs and coordinates were collected every 10 ps. A control simulation (without Gd@C_82_(OH)_22_ molecules) was also performed in the same simulation conditions.

### 2.2. Potential of Mean Force (PMF)

The umbrella sampling technique [55,56,57] was used to compute the potential of mean force of Gd@C_82_(OH)_22_ along the perpendicular direction of the membrane surface. The distance (d) to its binding site was restrained at a reference distance (d_0_) with a harmonic force F = k × (d – d_0_), where k is the force constant. A harmonic force of 2000 kJ/mol/nm^2^ was used in the Z direction (the perpendicular direction to membrane surface). The spacing of the sampling windows was set at 0.1 nm. At each window, a system was simulated for 6 ns, i.e., a 1-ns equilibration plus 5-ns production run. The free energy profiles were generated by Weighted Histogram Analysis Method (WHAM) [58,59].

## 3. Results and Discussion

**Figure 2:**
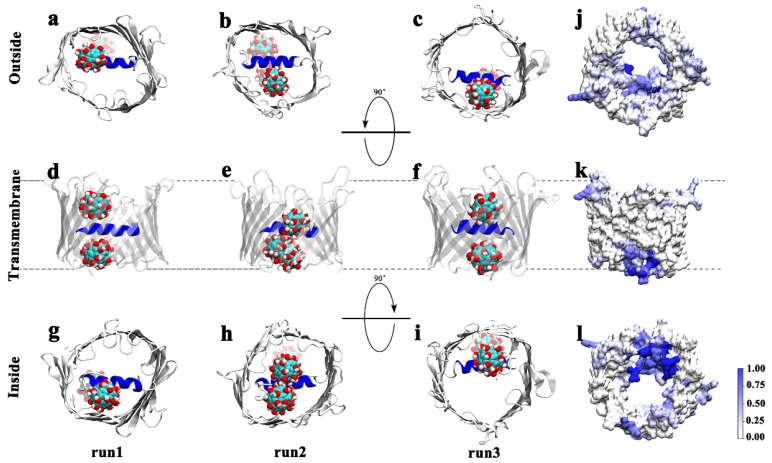
(**a**–**i**) Final snapshots taken from representative runs 1–3 obtained at the end of hundred-nanosecond MD simulations. The location of the membrane is indicated by the black dashed line in the middle row (**d**–**k**). For each run, the binding site of Gd@C_82_(OH)_22_ in the hVDAC1 is shown from the side view (middle), the top view from the outside of the membrane (top, to show the OM penetration), and the bottom view from the inside of the membrane (bottom, to show the IM penetration). (**j**–**l**) Average contact probability of Gd@C_82_(OH)_22_ on the hVDAC1 surface statistics obtained from the three runs.

Before investigating the interactions between Gd@C_82_(OH)_22_ and hVDAC1, a control MD simulation was conducted to test the structural stability of hVDAC1 in the membrane. Here, the NMR solution structure of hVDAC1 was inserted into a preformed dimyristoyl phosphatidylcholine (DMPC) lipid bilayer (Figure 1). After one hundred nanoseconds of simulation time, we observed no structural alterations in the conformation of hVDAC1 (Supplementary Materials Figure S1). After 50 ns of simulation time, the root-mean-square deviation (RMSD) of the hVDAC1 backbone equilibrated at around 0.32 nm, indicating the stability of the hVDAC1 protein embedded in the DMPC bilayer (Figure S2a). Following this finding, we simulated the interaction between Gd@C_82_(OH)_22_ molecules and hVDAC1 using the representative initial configuration in Figure 1c, where ten Gd@C_82_(OH)_22_ molecules were randomly distributed around the channel. Nine independent simulations (runs 1–9) were performed and each one lasted for at least 100 ns. In each run, the RMSDs of the hVDAC1 protein stabilized at around 0.30–0.40 nm after 60 ns simulations (Figure S2b). We observed that Gd@C_82_(OH)_22_ consistently penetrates the lumen of the hVDAC1, adopting different binding poses and binding kinetics (Figure S3a). Three of the nine runs (run 1–3) were used as representative to demonstrate the details as follows: Figure 2a–i delineates the last snapshots obtained for Gd@C_82_(OH)_22_ binding to the hVDAC1 protein from the three representative simulation runs. Here, we observed that at least two Gd@C_82_(OH)_22_ molecules are retained inside the β-barrel in runs 1–3. The simulation trajectories indicate that the Gd@C_82_(OH)_22_ molecules can enter the pore either from the outside of the membrane (OM) or the inside of the membrane (IM). After penetrating the pore, most of the Gd@C_82_(OH)_22_ molecules form contacts with the inner helix of hVDAC1. The Gd@C_82_(OH)_22_ molecules that penetrate from the OM are often also stabilized by the inner wall of the β-barrel; while those penetrating from the IM are often stabilized by the N-terminal loop of hVDAC1. The simulations also show that the inner helix of hVDAC1 precludes Gd@C_82_(OH)_22_ from permeating through the pore during the time frame studied.

To provide insights into the binding area, the average contact ratio of Gd@C_82_(OH)_22_ was mapped onto the surface structure of the hVDAC1 using the last 20 ns trajectories of the three representative runs (Figure 2j–l). A contact is counted when a heavy-atom pair from a target residue and Gd@C_82_(OH)_22_ is within 0.5 nm. The average contact ratio is the number of contacts for a residue normalized to the total number of contacts over the size trajectories. The region with the highest frequency of contacts was localized to the N-terminal loop and the inner helix. Specifically, the highest frequency of contacts was located at the N-terminal loop, corresponding to the structure that we observed to trap Gd@C_82_(OH)_22_ molecules that had diffused into the pore from the IM. Additionally, the internal wall of the β-barrel, as well as the loops between the β-sheets, also contribute to contacts with the Gd@C_82_(OH)_22_ molecules.

### 3.1. Binding Interactions and Kinetics of Gd@C_82_(OH)_22_ Entering the Lumen of hVDAC1 from the OM

**Figure 3:**
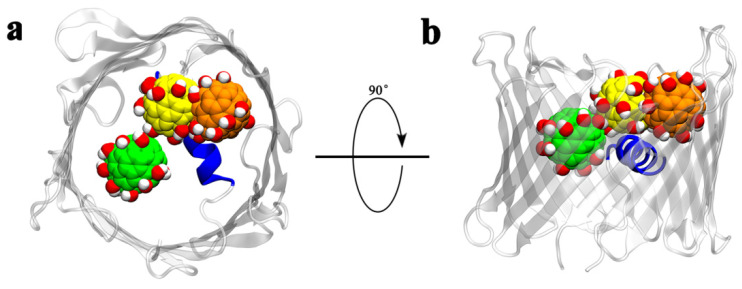
Superimposition of the three representative runs based on the backbone of hVDAC1 to show the relative positions of the Gd@C_82_(OH)_22_ molecules that penetrated from OM. Top view from OM is shown in (**a**) and side view in (**b**).

Next, we assessed each of the penetration pathways from OM and IM separately. When Gd@C_82_(OH)_22_ molecules entered the lumen of the hVDAC1 pore from the OM, three classes of binding sites were sampled by the MD simulations (Figure S3b): (1) Gd@C_82_(OH)_22_ positions on the N-terminal of the inner α-helix and interacts with the residues across β-strands 9–14 (run 1, Figure 2a); (2) the Gd@C_82_(OH)_22_ locates between side of the inner α-helix and the internal surface of the β-barrel (strands 3–7, run 2, Figure 2b); (3) the Gd@C_82_(OH)_22_ stays above the inner α-helix and interacts with residues in β-strands 12–16 (run 3, Figure 2c).

Based on the superimposition of the three binding configurations (Figure 3), we determined that Gd@C_82_(OH)_22_ could bind different sites of the porin. The deepest insertion occurred in run 2, in which Gd@C_82_(OH)_22_ squeezed into the interspace between the helix and inner wall of the β-barrel. This interaction is likely influenced by the Gd@C_82_(OH)_22_ cluster below that localizes below this position following penetration from the IM (Figure 2b). In runs 1 and 3, Gd@C_82_(OH)_22_ inserted to a similar depth in the pore, slightly shallower than that observed in run 2. However, in runs 4, 5, 7, and 8, we observed binding poses that suggested an even shallower insertion. In some cases, principal contact sites came exclusively from residues in the β-strands and not the inner α-helix (Figure S3). The highly flexible loops on the edge of porin tend to capture Gd@C_82_(OH)_22_ molecules and prevent them from diffusing further, which partly explains why Gd@C_82_(OH)_22_ molecules repeatedly bind to sites with extra-membrane loops nearby.

**Figure 4:**
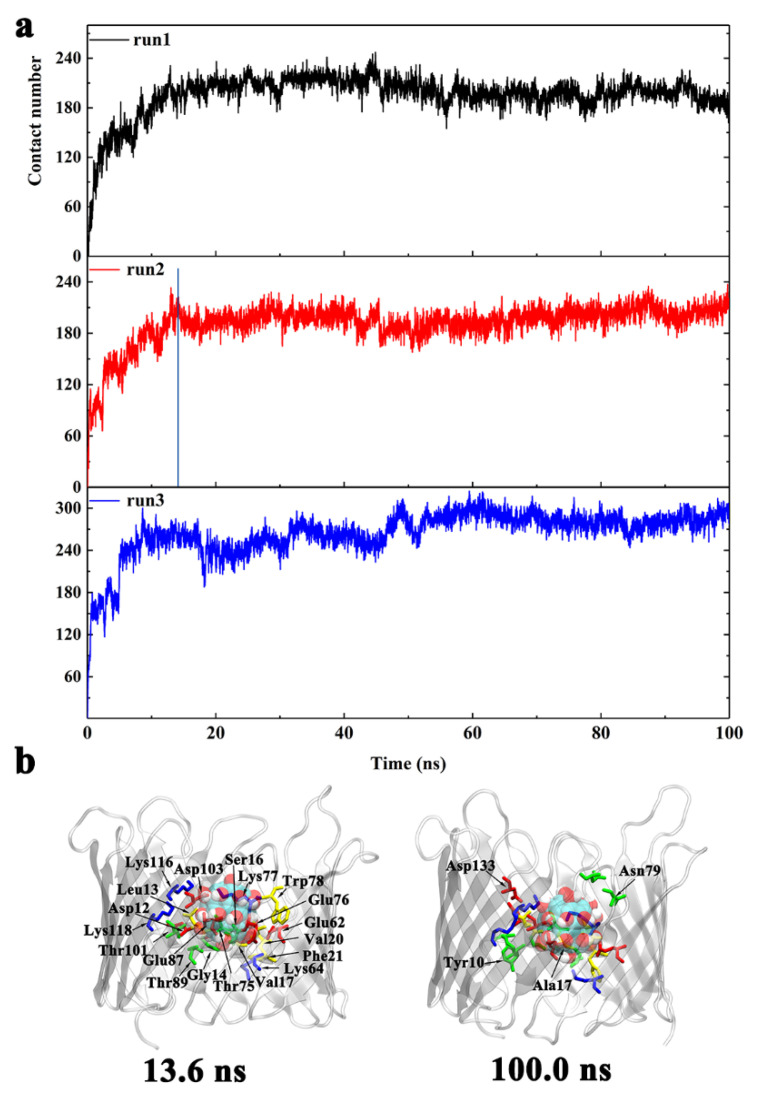
(**a**) The evolution of the total number of atomic contacts between hVDAC1 and Gd@C_82_(OH)_22_ molecules that penetrated the channel from OM in runs 1–3. The blue line represents key time points of the protein–nanoparticle interactions. (**b**) The binding conformations of Gd@C_82_(OH)_22_ to hVDAC1 at key time points corresponding to (**a**) as well as the final conformation. The key residues that contact Gd@C_82_(OH)_22_ are labeled and colored according to the residue type.

In order to understand the underlying binding kinetics involved in these interactions, we calculated the evolution of the total number of atomic contacts for the three systems (Figure 4). An atomic contact is calculated based on a distance cutoff of 0.6 nm between Gd@C_82_(OH)_22_ and the channel protein. The evolution of total atomic contacts increased over time until the system reached an equilibrium state (Figure 4a). At equilibrium, the hVDAC1 contributed to 180–200 contacts of Gd@C_82_(OH)_22_ molecules, stabilizing it at the relevant binding sites.

Here, we used run 2 as representative to demonstrate the analysis of the binding kinetics. Along the evolution of the total contact number, key snapshots were selected and shown with highlighted critical contact residues in Figure 4b. The penetration pathway of Gd@C_82_(OH)_22_ into the porin can be separated into two phases:

(1) From t = 0 to 13.6 ns, Gd@C_82_(OH)_22_ promptly entered into the porin with the total number of atomic contacts sharply increasing to ~190. At this stage, Gd@C_82_(OH)_22_ interacted with residues D12, L13, G14, S16, V17, V20, F21, E62, K64, E87, T89, T101, D103, K116, and K118 (Figure 4b). Of these residues, D12 to F21 are located at the inner helix, comprising 50% of the helical residues. Statistics of the contact residue types showed there are nine charged (five acidic, four basic), five hydrophilic, and five hydrophobic/aromatic residues, indicating the diversity of residues that Gd@C_82_(OH)_22_ can interact with in the protein tertiary structure. Gd@C_82_(OH)_22_ molecules contain both abundant hydroxyl groups and exposed aromatic rings on the surface; therefore, it has the capacity to form hydrogen bonds and hydrophobic interactions with local surrounded protein residues, making it a ‘versatile’ molecule.
(2) From t = 13.6 to 100 ns, the total contact number reached a long plateau and fluctuated around 200. At this stage, the Gd@C_82_(OH)_22_ molecule was observed to interact with four additional residues: Y10, A17, N79, and D133 (Figure 4b). Of the four residues, Y10 and A17 are from the inner helix, indicating a deeper insertion of Gd@C_82_(OH)_22_ into the lumen of the hVDAC1. Now, Gd@C_82_(OH)_22_ is positioned at the interspace of the helix and β-barrel and fully blocks the hVDAC1 porin. The RMSD of hVDAC1 backbone stabilized at around 0.35 nm during this stage (Figure S2), implying that an equilibrated binding mode had formed between Gd@C_82_(OH)_22_ and the protein interface.

### 3.2. Binding Interactions and Kinetics of Gd@C_82_(OH)_22_ Entering the Lumen of hVDAC1 from the IM

**Figure 5:**
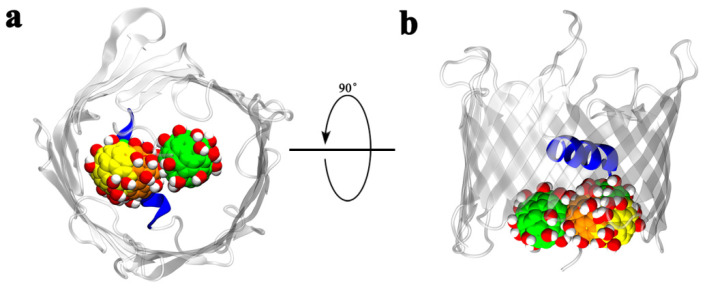
The superimposition of the three representative runs based on the backbone of hVDAC1 to show the relative positions of Gd@C_82_(OH)_22_ molecules that penetrated the pore from the IM. Top view from IM is shown in (**a**) and side view in (**b**).

In addition to diffusion from outside of the membrane (OM), our simulations show that Gd@C_82_(OH)_22_ can penetrate into the lumen of hVDAC1 from the inside of the membrane (IM, Figure 2 and Figure S3). In most of the nine simulations we performed, Gd@C_82_(OH)_22_ molecules interacted with the N-terminus loop of the hVDAC1. Superimposition of the final conformations that we captured clearly showed that the Gd@C_82_(OH)_22_ molecules from three independent runs occupied the same site; only one Gd@C_82_(OH)_22_ in run 2 located to an alternative binding site (likely as a result of two molecules penetrating the lumen of hVDAC1 in this simulation; Figure 5). Thus, we can conclude that the N-terminus of hVDAC1 plays a key role in attracting and stabilizing Gd@C_82_(OH)_22_ molecules. This conclusion is supported by analysis of the surface map of contact probabilities, in which the N-terminal region renders the highest contact probability in dark blue (Figure 2l).

**Figure 6:**
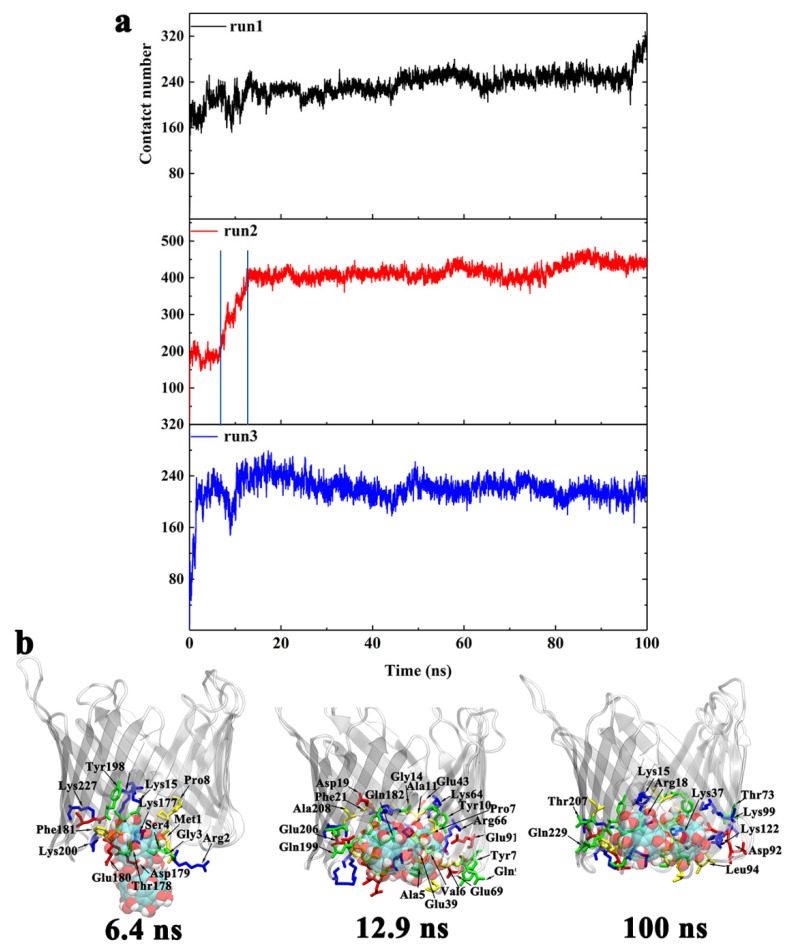
(**a**) The evolution of the total number of atomic contacts between hVDAC1 and Gd@C_82_(OH)_22_ molecules that penetrated from the IM for runs 1–3. The blue line represents key time points of the protein–nanoparticle interactions. (**b**) The binding conformations of Gd@C_82_(OH)_22_ to hVDAC1 at key time points corresponding to (**a**) as well as the final conformation. The key residues that contact Gd@C_82_(OH)_22_ are labeled and colored according to the residue type.

The evolution of the total number of atomic contacts between Gd@C_82_(OH)_22_ and protein was calculated using the same protocol as the OM side (Figure 6). In runs 1 and 3, which have one Gd@C_82_(OH)_22_ inserted in the lumen, the total number of atomic contacts fluctuated around 240. In run 2, two molecules of Gd@C_82_(OH)_22_ permeated the lumen, increasing the total atomic contacts to around 430.

Similar to the analysis we performed when Gd@C_82_(OH)_22_ permeated hVDAC1 from the OM side, a representative run was used to demonstrate the binding kinetics of IM permeant molecules. Here, we selected run 2 to illustrate the dynamic process of two molecules of Gd@C_82_(OH)_22_ penetrating the porin. Key snapshots were selected according to the evolution of the total contact number (Figure 6b). The permeation pathway of Gd@C_82_(OH)_22_ into the porin can be separated into three phases:

(1) From t = 0 to 6.4 ns, a transient plateau was formed with total contact number staying at around 200, indicating a relatively stable conformation with one Gd@C_82_(OH)_22_ molecule contacting with the protein. The intimate contacts were formed between the molecule and M1, R2, G3, S4, P8, K15, R18, K177, T178, D179, E180, F181, Y198, K200, and K227. Of these residues, M1 to P8 are located on the N-terminus, K15 and R18 are located on the inner helix, and K177 to F181 are located at the loop connecting β-strand 11 and β-strand 12. At this time point, Gd@C_82_(OH)_22_ mainly interacted with the intracellular residues and had not fully entered the central pore.
(2) From t = 6.4 to 12.9 ns, the first Gd@C_82_(OH)_22_ inserted further; meanwhile, the second Gd@C_82_(OH)_22_ engaged in contacting with the protein. Accordingly, the total contact numbers sharply increased from 200 to 400. This increase corresponded to 21 additional residues forming contacts with Gd@C_82_(OH)_22_: A5, V6, P7, P8, Y10, A11, G14, D19, F21, E39, E43, K64, R66, E69, Y70, E91, Q93, Q182, Q199, E206, and A208. In this list, A5 to F21 comprise 42.8% of residues and are on the N-terminus and the inner helix, indicating a deeper insertion of Gd@C_82_(OH)_22_ into the pore of hVDAC1.
(3) From t = 12.9 ns to the end of the simulation time, the total contact number stabilized at around 430. At this stage, two molecules of Gd@C_82_(OH)_22_ fully blocked the pore of the channel. Ten additional residues formed contacts with Gd@C_82_(OH)_22_: K15, R18, K37, T73, D92, L94, K99, K122, T207, and Q229. The statistics of residue types showed that there are 14 hydrophobic, 14 hydrophilic, 10 basic, and 10 acidic residues that interact with the Gd@C_82_(OH)_22_ cluster in the final conformation, again indicating the amphiphilicity of the Gd@C_82_(OH)_22_ molecules that have the capability to contact a variety of amino acid residues.

### 3.3. Interaction Energy Calculations between hVDAC1 Protein and Gd@C_82_(OH)_22_

To compare the different interaction modes of Gd@C_82_(OH)_22_ with hVDAC1, the interaction energy was calculated for the complexes based on the last 20 ns of the trajectories for each system. For systems in which Gd@C_82_(OH)_22_ bound from OM, the average total interaction energies were −369.48 kJ/mol, −365.86 kJ/mol, and −392.86 kJ/mol for runs 1, 2, and 3, respectively (Figure S4a). The average total interaction energies in runs 1 and 2 are comparable and were both slightly less than that calculated for run 3. Decomposition of the total interaction energy into van der Waals and coulombic terms showed that for run 1, the van der Waals energy was −169.81 kJ/mol and the Coulombic energy was −199.68 kJ/mol; for run 2, the van der Waals energy was −133.23 kJ/mol and the Coulombic energy was −233.53 kJ/mol; for run 3, the van der Waals energy was −210.89 kJ/mol and the Coulombic energy was −181.97 kJ/mol.

For systems where Gd@C_82_(OH)_22_ bound from IM, the average total interaction energies were higher: −535.61 kJ/mol, −865.78 kJ/mol, and −334.15 kJ/mol for runs 1, 2, and 3, respectively (Figure S4b). Note that in the case of run 2, the calculation involved two Gd@C_82_(OH)_22_ molecules’ interaction with the protein, which generate around 2-times the average total interaction energy of runs 1 and 3. Decomposition of the total interaction energy into van der Waals and coulombic terms showed that for run 1, the van der Waals energy was −216.50 kJ/mol and the Coulombic energy was −319.11 kJ/mol; for run 2, the van der Waals energy was −401.58 kJ/mol and the Coulombic energy was −464.20 kJ/mol; for run 3, the van der Waals energy was −166.66 kJ/mol and the Coulombic energy was −167.49 kJ/mol. Our analysis indicates that in most cases, the coulomb force dominates the interaction energy between hVDAC1 and Gd@C_82_(OH)_22_.

**Figure 7:**
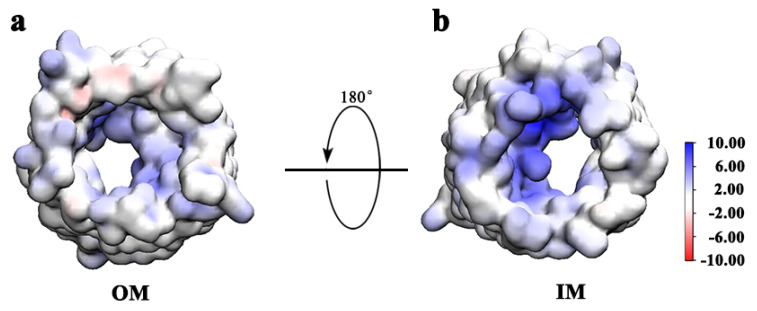
The electrostatic surface potential of hVDAC1 seen from OM side (**a**) and IM side (**b**). It is calculated using the Adaptive Poisson–Boltzmann Solver, APBS.

Although Gd@C_82_(OH)_22_ can penetrate the pore of hVDAC1 from IM and OM sides, both the interaction energies and the number of contacts formed are higher when Gd@C_82_(OH)_22_ permeates hVDAC1 from the IM. To understand the underlying mechanism, the electrostatic surface potential of hVDAC1 was calculated using a continuum electrostatic model (Adaptive Poisson–Boltzmann Solver, APBS) [60,61,62]. As presented in Figure 7, hVDAC1 exhibits a higher density of positive charges on the IM-facing surface than the OM-facing surface, particularly in the N-terminal and inner helical areas of the protein. Consistent with this finding, previous studies have elucidated that protein interactions occur predominantly with the negative-charged region of Gd@C_82_(OH)_22_ [27]. Briefly, the fullerenol cage of Gd@C_82_(OH)_22_ has a −3e negative charge, which is attributed to the encapsulated Gd^3+^ ion. Thus, a stronger nonspecific long-range electrostatic attraction exists between Gd@C_82_(OH)_22_ and the IM side of hVDAC1. It is noteworthy that the chance is equal for a Gd@C_82_(OH)_22_ molecule approaching the IM and OM sides of the porin in the current simulation setup; however, in the nine runs, we observed that more Gd@C_82_(OH)_22_ molecules entered hVDAC1 from IM. In the real cellular environment, the concentrations of Gd@C_82_(OH)_22_ between the IM and OM sides may differ as Gd@C_82_(OH)_22_ molecules would need to diffuse through the mitochondrial outer membrane to approach the IM side of the porin. Nevertheless, our data suggest that a Gd@C_82_(OH)_22_ molecule is thermodynamically more stabilized at the IM-surface of hVDAC1 than at the OM-surface. Next, we evaluated and compared the binding free energies for Gd@C_82_(OH)_22_ on each side of hVDAC1.

### 3.4. PMF Calculation of Gd@C_82_(OH)_22_ Interactions with hVDAC1 Protein

The interaction energies discussed above describe the enthalpy change between two entities in a biological system. However, it is more important to consider the binding free energy, which includes both the enthalpy and entropy contributions for a given binding event. Here, a potential of mean force (PMF) analysis was conducted to evaluate the binding free energies between Gd@C_82_(OH)_22_ and hVDAC1 at 300 K using an umbrella sampling technique. PMF calculations are a common approach for assessing and comparing the binding capacity of different interaction configurations [63,64,65,66,67,68,69]. Compared with the experimental measurements, the PMF calculations may give an overestimation of the free energy values; however, a good correlation is observed between the computational and experimental values [70]. Therefore, we performed PMF calculations for the Gd@C_82_(OH)_22_–hVDAC1 complexes by monitoring the relative free-energy change when we pulled the nanoparticles perpendicular to the membrane surface, i.e., the z direction.

**Figure 8:**
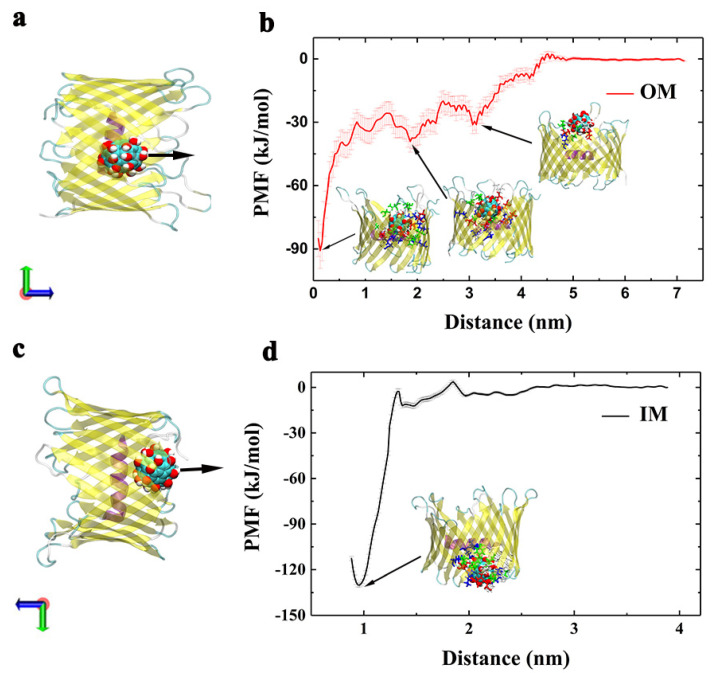
The binding free energies of Gd@C_82_(OH)_22_ on the hVDAC1 that permeated the channel from OM (**a**,**b**) and IM sides (**c**,**d**), respectively. The initial structures used in the PMF calculations are shown in panel (**a**,**c**); the arrows indicate the pulling directions. The structures that correspond to the energy wells are inset in the panels (**c**,**d**). Their contact residues in each structure are shown with stick and colored based on residue type.

PMF calculations were performed on representative binding configurations obtained from both the IM- and OM-surfaces (Figure 8). For the system bound from OM, the lowest binding free energy well was −90.78 kJ/mol, which refers to the most stable conformation formed in the MD simulations. Additionally, the second and third potential wells were also formed along the reaction coordinates, corresponding to −39.07 kJ/mol at z = 1.86 nm and −31.14 kJ/mol at z = 3.14 nm, respectively. The three conformations illustrated in Figure 8a correspond to the potential wells, respectively.

In contrast, only one potential well was formed in the system where Gd@C_82_(OH)_22_ interacted with hVDAC1 from the IM-surface, corresponding to PMF value −130.08 kJ/mol. The binding free energy for the configuration from IM was around 1.5 times greater than the OM-configuration. This difference is in accord with the higher total contacts number observed in the IM-configuration (Figure 4a and Figure 6a). Please note that the calculated free energy values for the IM-bound (−130.08 kJ/mol) or the OM-bound (−90.78 kJ/mol) likely do not reflect the true values. When Gd@C_82_(OH)_22_ is pulled along the z-direction to separate from hVDAC1, it forms contacts with the interwall and rim of hVDAC1 at some of the sampled windows, which may involve conformational changes of the protein that is not finely captured (or fully relaxed)—particularly the rim—leading to an overestimation of the binding free energies [65]. Nevertheless, it remains reliable that Gd@C_82_(OH)_22_ binding at IM-surface is stronger than that at OM-surface on account of the remarkable energy difference between them.

In addition to penetrating into the lumen of hVDAC1, we observed Gd@C_82_(OH)_22_ molecules binding to the rim of β-barrel, i.e., the nanomaterial can interact with the extracellular or intracellular loops that connect the strands of the β sheets (Figure S5). Some of the loops may contribute to a relatively stable interaction with Gd@C_82_(OH)_22_ molecules. For example, we observed in four of the nine runs that Gd@C_82_(OH)_22_ interacts with a nine-residue loop connecting β18 and β19. In particular, K269 located within this loop appears to play a role in stabilizing the interaction with Gd@C_82_(OH)_22_. However, a PMF calculation on a representative run to evaluate the binding strength of Gd@C_82_(OH)_22_ at this site demonstrated that the interaction energy was less (−25.80 kJ/mol) than the values we observed when Gd@C_82_(OH)_22_ interacted with the pore lumen of hVDAC1.

Our simulation data indicated the potential blockade of the hVDAC1 channel by Gd@C_82_(OH)_22_. To illustrate how this blockage might affect the transport of ATP, we first compared the complex of hVDAC1–Gd@C_82_(OH)_22_ with the cocrystallized structure of mouse VDAC1 with ATP (PDB code 4C69) [71] (Figure S6). Based on this analysis, it is clear that Gd@C_82_(OH)_22_ molecules can partially occupy the ATP binding site. In this binding configuration, the Gd@C_82_(OH)_22_ molecules localized to the center of the pore, resulting in a constriction that would limit the flux of ATP and other solutes through the channel. Second, it has been reported that ATP molecules utilize a number of distinct and interconnected pathways to get through the VDAC1 [71], and the process involves a series of pore-lining basic residues—particularly K12, R15, and K20 in N-terminal helix and R218 in outer mouth [71,72,73,74,75,76,77]—which play a key role in ATP transportation. Thus, we check the average contact probabilities of these residues with Gd@C_82_(OH)_22_ based on the three representative runs (Table S1). All of these residues are conserved between mouse and human VDAC1. Over half of these basic residues contribute to more than 30% of the contact with the Gd@C_82_(OH)_22_ molecules in our systems, also implying a possible interference of ATP flux by the nanomaterial.

## 4. Conclusions

Here, we employed MD simulations and free energy calculations to study the interactions between Gd@C_82_(OH)_22_ and hVDAC1—a vital porin embedded in the mitochondria outer membrane. In each of the nine independent simulations, we observed that Gd@C_82_(OH)_22_ molecules consistently penetrate the pore lumen of hVDAC1. Further, we determined that Gd@C_82_(OH)_22_ can penetrate the pore of hVDAC1 from both the OM and the IM sides. Penetration from OM resulted in multiple binding poses that were each sampled in the simulations. The most stable of these poses corresponds to interactions between Gd@C_82_(OH)_22_ and the inner helix and the inner wall of the β-barrel, resulting in a blockade of the hVDAC1 pore. Additional poses show that Gd@C_82_(OH)_22_ can interact mainly with the inner helix or the internal face of the β-barrel. These poses have lower interaction energies and could represent metastable states captured during the binding process.

Different from OM, when Gd@C_82_(OH)_22_ penetrated hVDAC1 from the IM, we observed a highly preferred binding pose that was common among the independent simulations. In this case, the binding site is shaped by the N-terminal loop, the inner helix, and inner wall of the β-barrel structure. This preferred binding mode is characterized by a higher number of contacts that we propose increases the stability of Gd@C_82_(OH)_22_, corresponding to higher binding free energies. Furthermore, in one of the independent runs, we observed that two molecules of Gd@C_82_(OH)_22_ penetrate into the lumen of hVDAC1, with one occupying the preferred binding site and the other equilibrating nearby. Together, the two Gd@C_82_(OH)_22_ molecules occlude the pore of the hVDAC1.

The Gd@C_82_(OH)_22_ molecule itself has unique structural characteristics, including multiple surface hydroxyl and aromatic groups, that facilitate interactions between the nanomaterial and both hydrophobic and hydrophilic residues in hVDAC1. Meanwhile, due to induction effect of the encapsulated Gd^3+^ ion, the carbon cage appears negatively charged, allowing for interactions with positive-charged entities via long-range electrostatic attraction [12,25,27]. Inspection of the electrostatic surface potential of hVDAC1 supports electrostatic interactions between Gd@C_82_(OH)_22_ and the lumen of the protein, in particular, the IM-surface.

Analysis of binding free energies allows for estimates of the binding stability for biological complexes and/or comparisons in binding strength between different configurations—in this case, Gd@C_82_(OH)_22_ from OM and IM side bound to the hVDAC1. The penetration free energy of Gd@C_82_(OH)_22_ from the OM side is lower than from the IM side (−90 kJ/mol, verses −130 kJ/mol), consistent with the above analysis of the binding sites and electrostatic matching.

In summary, our results provide an insight into the molecular interactions between Gd@C_82_(OH)_22_ and hVDAC1. The Gd@C_82_(OH)_22_ molecules can adopt multiple binding poses on hVDAC1, several of which would result in blockade of the channel pore. Given the key biological role of hVDAC1 in cellular physiology and tumorigenesis, interactions between Gd@C_82_(OH)_22_ and the channel are worthy of further, longer-term experimental investigations.

## Data Availability

Not applicable.

## Supplementary Materials

The following are available online at https://www.mdpi.com/article/10.3390/biom12010123/s1, Figure S1: The initial and final conformations of hVDAC1 in the absence of the Gd@C_82_(OH)_22_, Figure S2: The evolution of RMSD of hVDAC1 for the control run without Gd@C_82_(OH)_22_ (top) and averaged values over the nine runs with Gd@C_82_(OH)_22_ (bottom), Figure S3: (a) Last snapshots of the nine runs obtained from hundred nanoseconds MD simulations. The location of the membrane is indicated by the dash line. (b) Superimposition of all nine runs based on their last snapshots, Figure S4: The van der Waals, coulomb and total interaction energies between hVDAC1 and Gd@C_82_(OH)_22_ for (a) Gd@C_82_(OH)_22_ penetrated from OM and (b) Gd@C_82_(OH)_22_ penetrated from IM, Figure S5, The binding free energy of Gd@C_82_(OH)_22_ on the rim of hVDAC1, Figure S6: Top and side view of the superimposition between the complexes of hVDAC1+Gd@C_82_(OH)_22_ (in magenta) and mVDAC1+ATP (in blue), Table S1: Contact probabilities of some basic residues to Gd@C_82_(OH)_22_.
