# Supplementary material for: The Molecular Mechanism of Human Voltage-Dependent Anion Channel 1 Blockade by the Metallofullerenol Gd@C82(OH)22: An In Silico Study
Source: Biomolecules. 2022 Jan 12;12(1):123. doi: 10.3390/biom12010123 (PMC8773804; doi:10.3390/biom12010123)
Supplement: Supplementary file 1 [file biomolecules-12-00123-s001.zip › biomolecules-1503055-supplementary.pdf]

*biomolecules*

MDPI

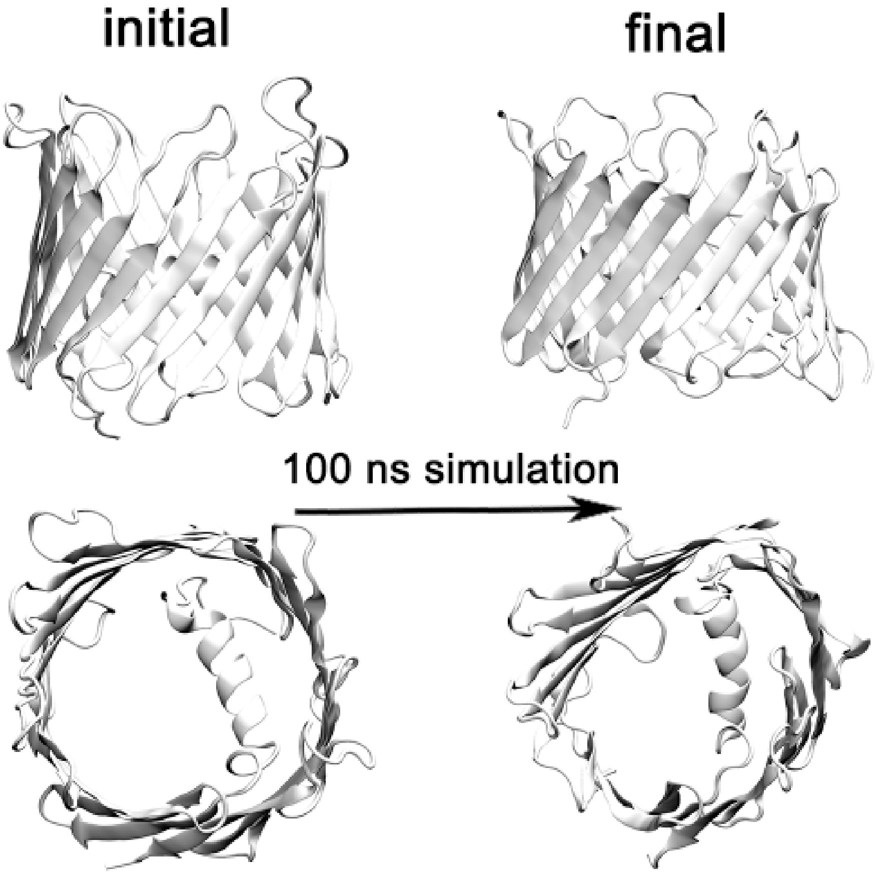


**Figure S1.** The initial and final conformations of hVDAC1 in the absence of the Gd@$C_{82}(OH)_{22}$.

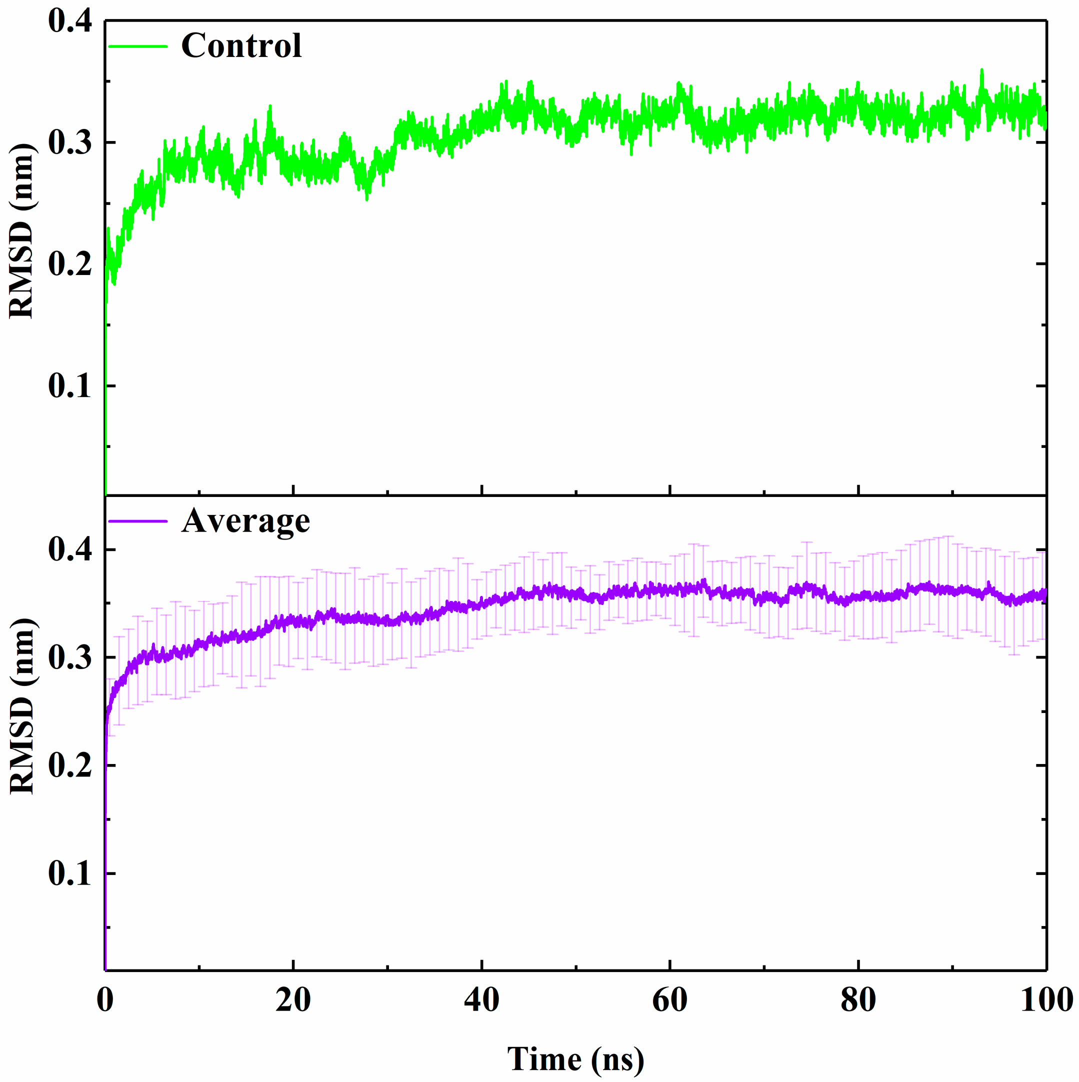


**Figure S2.** The evolution of RMSD of hVDAC1 for the control run without Gd@$C_{82}(OH)_{22}$ (top) and averaged values over the nine runs with Gd@$C_{82}(OH)_{22}$ (bottom).

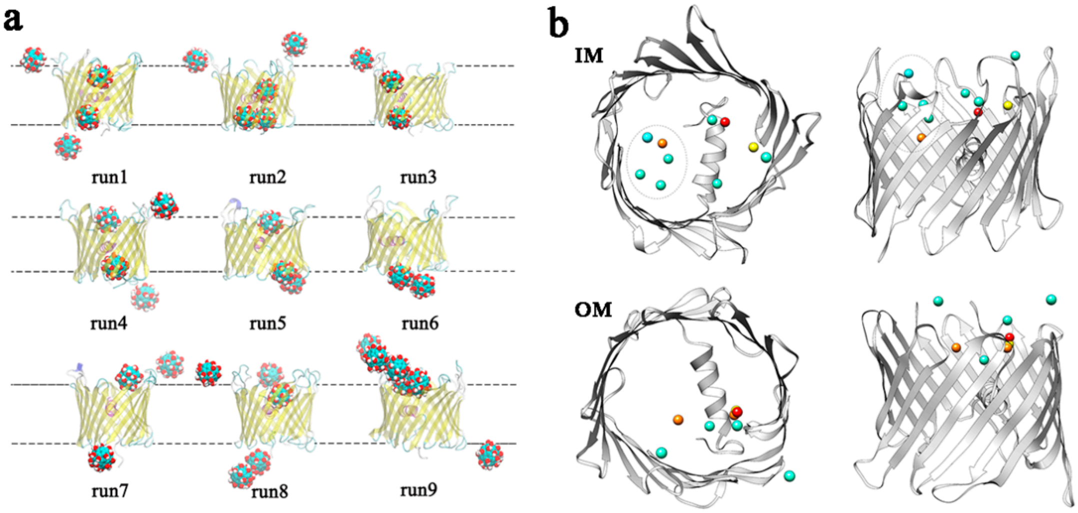


**Figure S3.** (a) Last snapshots of the nine runs obtained from hundred nanoseconds MD simulations. The location of the membrane is indicated by the dash line. (b) Superimposition of all nine runs based on their last snapshots. The encapsuled $Gd^{3+}$ ions were shown in sphere and the fullerene cages were hiding for clear view, of which the $Gd^{3+}$ ions from representative run 1, 2 and 3 were shown in red, orange and yellow, respectively. Runs 1-3 are selected as representative due to factors: 1) deeper penetration into the channel compared with other configurations, 2) their different positions at XY plane (configurations with similar XY-positions and different Z positions are regarded as one cluster), and 3) sampled more than once in the nine runs (referring to their XY-plane coordinates). The dash-circle is the cluster composed of run 2, 4, 5, 7 and 8 for the OM-surface binding. Run 2 (in orange) has the deepest insertion in this cluster. It was also selected to conduct PMF calculations. When pulled it along Z-direction, basically it may go through the configurations that are similar to the other four runs. The IM-surface binding use run 1 (in red) as representative to conduct PMF calculations as this site is highly preferred that sampled three times in the nine runs.

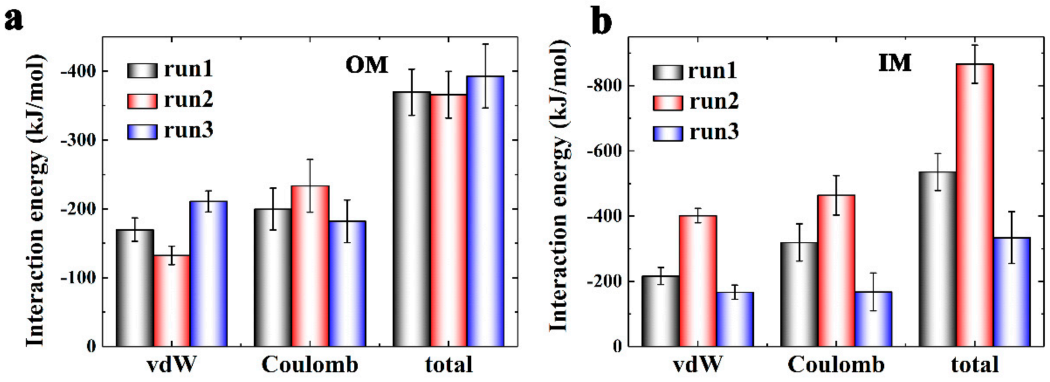


**Figure S4.** The van der Waals, coulomb and total interaction energies between hVDAC1 and Gd@$C_{82}(OH)_{22}$ for (a) Gd@$C_{82}(OH)_{22}$ penetrated from OM and (b) Gd@$C_{82}(OH)_{22}$ penetrated from IM. The calculation is based on the last 20 ns of the simulation data.

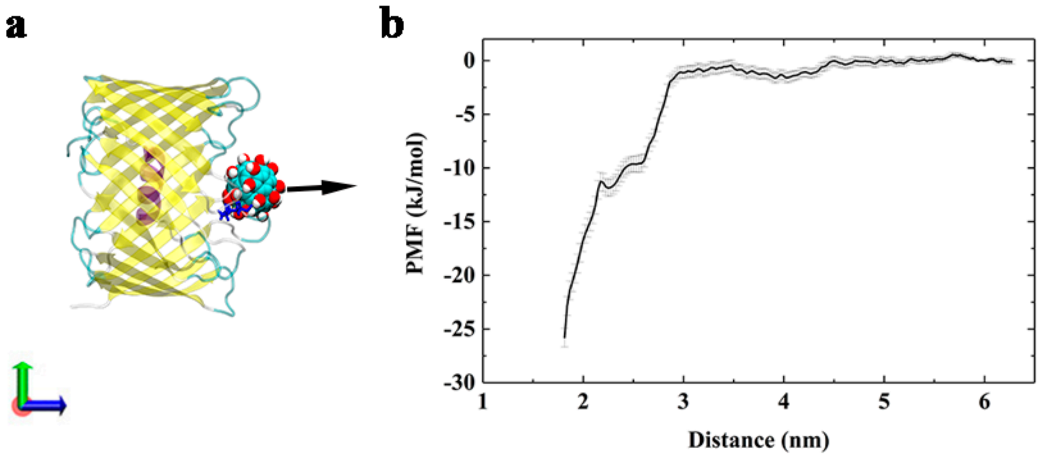


**Figure S5.** The binding free energy of Gd@$C_{82}(OH)_{22}$ on the rim of hVDAC1. The key contact residue K269 is shown with stick and colored based on the residue type.

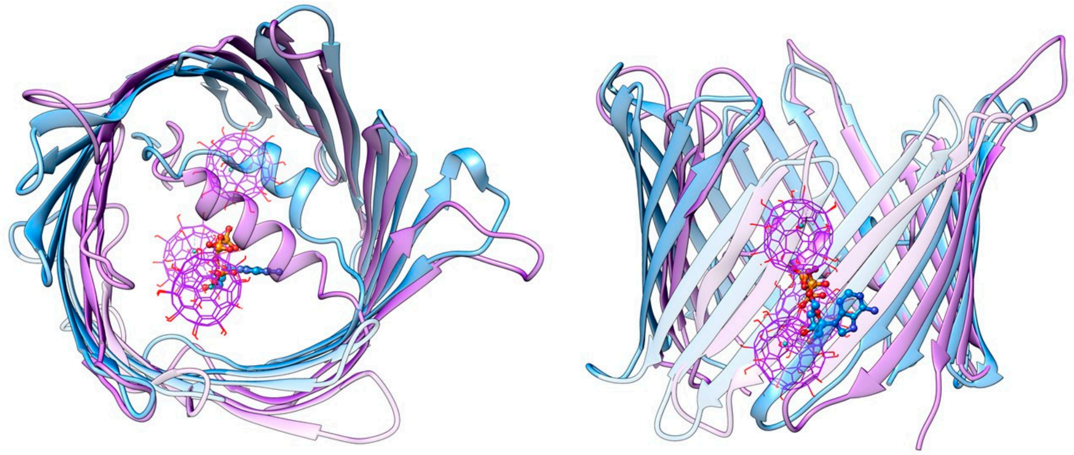

**Figure S6.** Top and side view of the superimposition between the complexes of hVDAC1+Gd@$C_{82}(OH)_{22}$ (in magenta) and mVDAC1+ATP (in blue). Here we used the configuration in run 2 to make the comparison as it is the most tightly bound complex obtained from the MD simulations. The Gd@$C_{82}(OH)_{22}$ molecules are shown in lines and the ATP is shown in ball and stick.

**Table S1.** Contact probabilities of some basic residues to Gd@$C_{82}(OH)_{22}$.

| Residue Number hVDAC1 | Residue Number Corresponding to mVDAC1 | Contact Probability to Gd@$C_{82}(OH)_{22}$ | Ref. |
|---|---|---|---|
| **K15** | K12 | 0.73 | [71,72,75-77] |
| **R18** | R15 | 1.00 | [71,72,75-77,] |
| **K23** | K20 | 0.01 | [71-73,75-77] |
| **K35** | K32 | 0 | [75] |
| **K37** | K34 | 0.23 | [75,77] |
| **K56** | K53 | 0 | [71,75,76] |
| **K64** | K61 | 0.17 | [75,77] |
| **R66** | R63 | 0.33 | [71,75] |
| **R96** | R93 | 0 | [71,75,77] |
| **K99** | K96 | 0.26 | [75] |
| **K112** | K109 | 0 | [71] |
| **K116** | K113 | 0.33 | [71] |
| **K118** | K115 | 0.12 | [71,72] |
| **K122** | K119 | 0.35 | [72,75,77] |
| **R142** | R139 | 0.33 | [71,75] |
| **K164** | K161 | 0.33 | [71] |
| **R166** | R163 | 0.33 | [71] |
| **K177** | K174 | 0.94 | [71] |
| **K203** | K200 | 0.18 | [75] |
| **R221** | R218 | 0.67 | [71,75,77] |
| **K227** | K224 | 0.43 | [75] |
| **K269** | K266 | 0.96 | [75] |

It has been reported ATP, AMP or phosphate ions may form contacts to the listed basic residues when crossing the VDAC1 channel. Related studies listed in the Reference column. The contact probabilities are calculated based on the last 20 ns trajectories of the three representative runs 1–. The same calculation and data were used to generate Figure 2j-l. Contact probabilities over 0.3 are highlighted in red.
